# The prevalence and impact of tension-type headache in school-aged children in Iran

**DOI:** 10.3389/fneur.2023.1259624

**Published:** 2023-09-14

**Authors:** Mansoureh Togha, Elham Jafari, Zhale Salami, Koorosh Kamali, Hadis Mirzaee Godarzee, Mohadeseh Mirzaee Godarzee, Sanaz Bavarnegin

**Affiliations:** ^1^Headache Department, Iranian Center of Neurological Research, Neuroscience Institute, Tehran University of Medical Sciences, Tehran, Iran; ^2^Department of Molecular Genetics, Faculty of Biological Sciences, Kharazmi University, Tehran, Iran; ^3^School of Public Health, Social Determinants of Health Research Center, Zanjan University of Medical Sciences, Zanjan, Iran; ^4^Medical Doctor, Tehran University of Medical Sciences, Tehran, Iran; ^5^Department of Molecular Genetics, Faculty of Biological Sciences, Tarbiat Modares University, Tehran, Iran

**Keywords:** children, adolescents, tension-type headache, study conducted in schools, quality of life

## Abstract

**Background:**

Tension-Type Headache (TTH) is regarded as the third most prevalent disorder worldwide, prompting children to seek medical attention. Our objective is to investigate the prevalence of TTH among students aged 6 to 18 years in various geographical regions of Iran, while also assessing the impact of headaches on their quality of life.

**Methods:**

Employing a cross-sectional survey, we have carefully distributed self-completed structured questionnaires to students in 121 meticulously selected schools throughout the country, ensuring the representation of its diverse population.

**Results:**

Among the 2,958 potential participants, we have included a total of 2031 individuals in our study. This comprises 57.3% children and 42.7% adolescents, with 50.02% being males and 49.97% females. Specifically, we have examined 950 subjects with TTH and 1,081 individuals without any form of headache. TTH was diagnosed in 32.1% of the participants. Notably, we have observed a significant difference in the average age between the TTH subjects and those without headaches. Participants without headaches were more likely to be enrolled in primary schools, while those diagnosed with TTH predominantly attended high schools. We found no significant relationship between urban–rural areas or different geographic regions and the prevalence of TTH or its subtypes. Phonophobia was commonly associated with TTHs. Lastly, the mean quality-of-life score was highest for individuals without headaches, followed by those with low frequency episodic TTH, high frequency episodic TTH, and chronic TTHs. There was also a significant relation between headache severity and quality of life scores.

**Conclusion:**

The significant prevalence of TTH in children and adolescents and its adverse impact on the daily activities of individuals underscore the utmost importance of accurate diagnosis and efficient management.

## Introduction

Tension-type headache (TTH) is the most prevalent disorder and the third most common disease worldwide for which children seek medical attention (1). Headaches in childhood hold significant importance as they can have an impact on a child’s education and lead to unfavorable outcomes for both children and their families, including school absenteeism and limited participation in school activities. These effects can ultimately result in a decline in the quality of education and future life prospects.

Furthermore, the avoidance of physical and academic activities is associated with conditions such as an increased risk of depression and reduced quality of life during adolescence and adulthood (2). Therefore, it is of utmost importance that TTH is not underestimated or misdiagnosed. However, diagnosing tension headaches, particularly in young children, poses a challenge as they may not be able to articulate the recognized characteristics of pain and accompanying symptoms.

Several factors, including both central and peripheral factors, such as pain sensitivity and modulation, biochemical mechanisms, emotional conflict and psychosocial stress, improper function of neck and shoulder muscles, and environmental influences, are involved in the development of TTHs (3, 4).

In the case of most children, tension-type headaches are typically triggered by stress-inducing situations and anxiety (5). Therefore, it is crucial to evaluate psychological stress factors and assess the effectiveness of different therapeutic approaches in managing tension-type headaches in children (6). It should be noted that epidemiological studies on headaches have utilized various definitions and classifications, which can pose challenges when comparing the data (7). The international classification of headache disorders(ICHD3) has designed criteria to diagnose TTH and its subtypes and to distinguish them from other headaches, including migraine (8).

Numerous genetic and epigenetic factors may potentially contribute to the presence and intensity of tension-type headaches in children. These factors encompass individual, familial, environmental, and geographical aspects (9). The specific genes that are causative of TTH are unknown (1). Furthermore, environmental factors, including stress, nutrition, and behavioral habits, can also exert an influence on the occurrence of tension-type headaches in children (10). Anxiety, depression and sleep disturbances are more common in individuals with TTH and are associated with the frequency and severity of TTH attacks (1). The purpose of this survey is to examine the prevalence of TTH among students residing in various geographical regions of Iran and to evaluate the headaches characteristics and possible influencing factors in episodic and chronic tension headaches, while also assessing the impact of headaches on the quality of life.

## Materials and methods

In a comprehensive cross-sectional survey, conducted from 1 December 2018 until 31 March 2019, meticulously crafted questionnaires were administered to pupils aged 6–18 during their school hours in 121 carefully selected schools from urban and rural areas of 13 of Iran’s 31 provinces, ensuring a representative sample that encompasses its diverse population in terms of geographical, genetic, cultural and socio-economic diversities. We selected 36 schools from the south of Iran, 41 schools from the north of Iran, 17 schools from the west, 13 schools from the east and 14 schools from the central parts of the country, including both urban and rural areas of each part. Thorough explanations regarding the study and its objectives were provided to the participants or their parents, and upon their agreement to partake in the study, they were kindly requested to complete the informed consent form. The study received the esteemed approval of the Ethics Committee of the Iranian Center of Neurological Research, under the ethical code IR.TUMS.NI.REC.1397.211.

The questionnaires were completed by the students under the supervision of either the investigators or their parents. In order to ensure that the younger children or those who had difficulty reading understood the questions and provided appropriate responses, assistance was provided.

The patient demographic data, including age, sex, geographic region, and educational level, were carefully recorded. Additionally, information was collected regarding various headache-related features. This included the location of the headache, its duration (less than 1 h, 1–2 h, 2–4 h, or more than 4 h), intensity, quality (throbbing or pressure-type), frequency, associated symptoms (nausea, vomiting, photophobia, and phonophobia), aggravation of the headache by physical activity, and the number of abortive medications used for headache treatment.

The diagnosis of TTH or another headache subtype was confirmed by neurologist/headache specialists according to ICHD3 criteria (8). Patients with other headache subtypes were excluded from the study. Tension-type headache was further divided to low frequency episodic TTH (LFET) (occurring <8 days/month), high frequency episodic TTH (HFET) (occurring 8–14 days/month) and chronic (occurring ≥15 days/month). The classification used for diagnosis of episodic versus chronic tension headache in our study was in accordance to ICHD-3 suggested criteria. The subdivision of episodic TTH to LFET and HFET is a suggestion by the authors taken from the term of high frequent episodic migraine used in the previous studies (11–13). It was to differentiate the characteristics and burden of the disease related to the headache frequency.

We used the Persian translation of the Headache-Attributed Restriction, Disability, Social Handicap and Impaired Participation (HARDSHIP) structured questionnaire for children and adolescents for assessing headache subtype, impact and quality of life (14).

### Statistical methods

Quantitative variables with a normal distribution have been expressed as the mean and the standard deviation (SD). The chi-square test was used to compare proportions for categorical variables between the case and control groups. The independent two-sample t-test was used for comparison of means. *p*-values of less than 0.05 were considered statistically significant. SPSS 24 (IBM Corp., Armonk, New York, USA) was used for all statistical analyses.

## Results

2,958 students aged 6–18 years from 121 urban and rural schools across the country participated in the study. A total of 2031 participants (children 1,163 (57.3%), adolescents 868 (42.7%); males 1,016 [50.02%], females 1,015 [49.97%]) were included in the study, 1,081 subjects without headache and 950 subjects with TTH. A total of 927 subjects were excluded from the study, including 809 students with migraine and 118 subjects due to the diagnosis of other headache disorders or incomplete filling of the questionnaires. TTH was diagnosed in 32.1% of the participants, including 873 individuals (29.5%) with LFET, 58 (1.9%) with HFET, and 19 (0.6%) with chronic TTH.

The average age of the participants was 11.8(3.3) (Mean ± SD) years, with an equal distribution of males and females. The selection process ensured representation from five different geographical regions of Iran, namely North (37.6%), East (10.9%), West (13.3%), Central (16.4%), and South (21.5%). These regions were further divided into urban (79.2%) and rural (20.9%) areas to capture a diverse sample. The educational level of the participants was primary school in 42.7% and high school in 57.3% of them.

There was a significant difference between the mean age of TTH subjects and those without headache (*p* < 0.001). The mean age for each type of tension headache also differed significantly, with chronic tension headaches having the highest mean age. The proportion of males and females did not differ significantly across the groups.

Regarding the academic level, there was a significant difference between TTH and no headache groups. Subjects without headache were more likely at the primary school level, while more individuals with TTH were at high school level. In addition, chronic tension headaches were significantly more prevalent among high school students. Specifically, 84.2% of the individuals with chronic TTH and 75.9% of those with HFET were high school students.

We found no significant relationship between urban–rural areas or different geographic regions and the prevalence of TTH or its subtypes. Table 1 shows the baseline characteristics of the participants.

**Table 1:** Baseline characteristics of participants in case and control groups.

Variables		Tension type headache			Subjects without headache	*p* value^α^	*p* value^β^	Total
		Low frequency	High frequency	chronic				
Number		873	58	19	1,081			2031
Age mean (SD)		12.9 (2.9)	14.2 (2.3)	14.7 (1.9)	10.82 (3.404)	<0.001	<0.001	11.8(3.3)
Gender Female number (%)		455 (52.2%)	32 (55.2%)	11 (57.9%)	517 (47.8%)	0.809	0.188	1,015(50%)
Academic level	Primary School	384 (43.9%)	14 (24.1%)	3 (15.8%)	763 (70.6%)	0.001	<0.001	1,164(57.3%)
	High School	490 (56.1%)	44 (75.9%)	16 (84.2%)	318 (29.4%)			868(42.7%)
Region	Urban	697 (79.8%)	48 (82.8%)	16 (84.2%)	840 (77.7%)	0.883	0.293	1,601(79.2%)
	Rural	167 (19.2%)	10 (17.2%)	3 (15.8%)	241 (22.3%)			421(20.8%)
Geographic area	North	331 (37.9%)	19 (32.8%)	7 (36.8%)	407 (37.7%)	0.360	0.013	764(37.7%)
	East	78 (8.9%)	4 (6.9%)	2 (10.5%)	138 (12.8%)			222(10.9%)
	West	110 (12.6%)	4 (6.9%)	1 (5.3%)	156 (14.4%)			271(13.4%)
	Center	139 (15.9%)	14 (24.1%)	6 (31.6%)	175 (16.2%)			334(16.5%)
	South	215 (24.6%)	14 (29.3%)	3 (15.8%)	205 (19.0%)			437(21.5%)

α *p* value is calculated among tension type headache (three groups).

β *p* value is calculated among all categories (four groups).

### Headache characteristics

Participants consistently described tension headaches as predominantly pressing type, affecting both sides of the head and worsened with physical activity. Low frequent TTHs had shorter durations and lower severity and were more likely to occur on one side than high frequent and chronic ones. Nausea, vomiting, and photophobia were not commonly reported symptoms, but 78.1% of our patients experienced phonophobia. LFETs were less likely to be associated with nausea and photophobia compared to high frequent and chronic headaches.

Low frequency TTHs had also a lower mean number of analgesic medication use, missed school days, and missed work days than high frequent and chronic ones (Table 2). Pupils with chronic TTH, missed a mean of 1.58 school days during the preceding 4 weeks. The corresponding rates were 0.28 for HFET and 0.13 for LFET. The mean number of days of analgesic use per month was 2.63 days for chronic TTH, 1.50 for HFET and 0.64 for LFET.

**Table 2:** Headache characteristics among subjects with tension-type headache.

Variable		Tension type headache				*p* value
		Total	Low frequency	High frequency	Chronic	
Duration	<1 h	621 (65.4%)	587 (67.2%)	26 (44.8%)	8 (42.1%)	<0.001
	1–2 h	250 (26.3%)	224 (25.7%)	20 (34.5%)	6 (31.6%)	
	2–4 h	59 (6.2%)	46 (5.3%)	9 (15.5%)	4 (21.1%)	
	>hours	20 (2.1%)	16 (1.8%)	3 (5.2%)	1 (5.3%)	
Severity	Not bad	755 (80.1%)	718 (82.8%)	32 (56.1%)	5 (26.3%)	<0.001
	Quite bad	166 (17.6%)	135 (15.6%)	19 (33.3%)	12 (63.2%)	
	Very bad	22 (2.3%)	14 (1.6%)	6 (10.5%)	2 (10.5%)	
Quality	Throbbing	242 (26.8%)	229 (27.7%)	10 (17.2%)	3 (15.8%)	0.120
	Pressing	660 (73.2%)	596 (72.1%)	48 (82.8%)	16 (84.2%)	
Laterality	One side	182 (19.5%)	175 (20.4%)	6 (10.5%)	1 (5.3%)	0.044
	Middle	353 (37.7%)	328 (38.3%)	18 (31.6%)	7 (36.8%)	
	Both sides	400 (42.8%)	354 (41.3%)	33 (57.9%)	13 (57.9%)	
Increased with Physical activity	No	676 (72.1%)	624 (72.3%)	41 (71.9%)	11 (61.1%)	0.583
	Yes	262 (27.9%)	239 (27.7%)	16 (28.1%)	7 (38.9%)	
Exercise avoidance	No	588 (62.7%)	542 (62.9%)	36 (62.1%)	10 (55.6%)	0.817
	Yes	350 (37.3%)	320 (37.1%)	22 (37.9%)	8 (44.4%)	
Nausea	No	665 (70%)	618 (71.1%)	32 (55.2%)	15 (78.9%)	0.025
	Yes	281 (30%)	251 (28.9%)	26 (44.8%)	4 (21.1%)	
Vomiting	No	871 (92.1%)	799 (91.9%)	53 (91.4%)	19 (100.0%)	0.577*
	Yes	75 (7.9%)	70 (8.1%)	5 (8.6%)	0	
Photophobia	No	657 (69.7%)	614 (70.9%)	32 (55.2%)	11 (57.9%)	0.023
	Yes	286 (30.3%)	252 (29.1%)	26 (44.8%)	8 (42.1%)	
Phonophobia	No	207 (21.9%)	196 (22.6%)	9 (15.5%)	2 (10.5%)	0.221
	Yes	739 (78.1%)	673 (77.4%)	49 (84.5%)	17 (89.5%)	
Headache days in the last month	Mean (SD)	2.7 (3.8)	1.84 (2.156)	10.44 (3.937)	17.11 (6.599)	< 0.001
Medication use in the last month	Mean (SD)	0.7 (1.8)	0.64 (1.425)	1.50 (2.522)	2.63 (5.747)	< 0.001
Missed school days in the last month	Mean (SD)	0.2 (1.1)	0.13 (0.682)	0.28 (0.914)	1.58 (5.719)	< 0.001
Missed work in the last month	Mean (SD)	1.1(2.5)	0.79 (1.629)	3.67 (4.915)	6.37 (7.537)	< 0.001
Parents’ missing work days	Mean (SD)	0.09 (0.5)	0.07 (0.484)	0.29 (0.937)	0.00 (0.000)	< 0.001

*Fisher exact test *p* value.

### Headache disability

The mean quality-of-life score was highest for individuals without headaches, followed by those with LFET, HFET, and chronic TTHs (*p* < 0.001), while there was no significant difference in quality-of-life scores between individuals with LFET and those without headaches (Table 3). LFETs had the lowest mean headache impact score compared to high frequent and chronic ones. Our analysis revealed no significant difference between males and females in quality of life or headache impact scores (Table 4).

**Table 3:** Quality of life scores of participants.

Study parameters	Quality of life score Mean (SD)	*p* value
Tension type headache		
Low frequency	40.9 (12.1)	0.271^α^ <0.001^β^
High frequency	38.9 (12.29)	
Chronic	37.3 (9.3)	
Subjects without headache	44.1 (11.6)	
Pain severity		
Not bad	41 (11.9)	0.106
Quite bad	39.3 (14.2)	
Very bad	43.3 (12.6)	
Geographic area		
North	43 (11.5)	<0.001
East	45.2 (10.7)	
West	39.8 (14.6)	
South	42.3 (12.1)	
Center	42.4 (10.9)	
Sex		
Male	42.5 (12.3)	0.788
Female	42.6 (11.7)	
Headache		
No	44.1(11.6)	<0.001
Yes	40.9 (12.1)	

α *p* value is calculated among tension type headache (three groups).

β *p* value is calculated among all categories (four groups).

**Table 4:** Headache impact scores of participants.

Study parameters	Headache impact score Mean (SD)	*p* value
Tension type headache		
Low frequency	58 (32.9)	<0.001
High frequency	81.7 (30.7)	
Chronic	72.2 (25.2)	
Pain severity		
Not bad	56.5 (33.1)	<0.001
Quite bad	69.6 (35.4)	
Very bad	83.3 (38.6)	
Geographic area		
North	57.8 (33.1)	0.339
East	65.1 (30.2)	
West	60.9 (36.5)	
South	58.3 (34.4)	
Center	62 (37.8)	
Sex		
Male	58.2 (34.1)	0.172
Female	61.2 (35)	

## Discussion

In this comprehensive analysis, we aim to shed light on the multifaceted nature of tension-type headaches and their profound impact on individuals’ daily lives among school-age children. Our findings indicated a high prevalence of TTH among the participants. Approximately 32.1% of the children reported experiencing this type of headache, including 29.5% with LFET, 1.9% with HFET, and 0.6% with chronic TTH. Togha, et al. (14) by a school-based study in Iran, reported a 1-year prevalence of 12.7% for TTH in children and adolescents. In the study of Kóbor, et al. (15) the overall 1-year prevalence of TTH was 29%, with a prevalence of 11, 17, and 1% for infrequent episodic, frequent episodic and chronic TTH. It should be noted that they used ICHD-3 beta for classification of episodic TTH subtypes.

Our study’s findings provide valuable insights into the heterogeneity of this condition by highlighting significant differences in variables such as headache duration, severity, quality, and location among different groups of individuals who suffered from tension headaches. Furthermore, our study explored the relationship between the occurrence of TTHs and age in children. The mean age of individuals with tension headaches varied significantly depending on the type of headache experienced. It seems that not only the prevalence of headache but also its phenotype and its impact on the quality of life undergo changes during the transition from childhood to puberty and adolescence. Numerous studies have been conducted to investigate the relationship between age and the prevalence of headaches among children (14). These studies provide evidence supporting the claim that the prevalence of headaches increases with age. For instance, Laurell et al. (16) conducted a study involving school-age children and found that TTHs are more prevalent in children aged 13 to 15, with girls reporting a higher incidence of headaches. Similarly, Anttila et al. (17) reported similar findings regarding the prevalence of TTHs among children.

In a study conducted by Togha et al. (14) in Iran, it was observed that the prevalence of headaches is higher among adolescents compared to children. This finding aligns with our own research, as well as the study conducted by Ayatollahi et al. (18), which demonstrated a positive correlation between age and the prevalence of headaches in children.

Furthermore, Poyrazog˘lu et al. (9) conducted a study in Turkey, which revealed that the prevalence of TTHs increases beyond the age of 15. Similarly, Yun Jin Jeong et al. (10) in Korea arrived at similar conclusions, noting that the prevalence of headaches in children rises as they progress from primary to elementary school. These findings provide additional evidence to support the notion that age plays a significant role in the prevalence of headaches among children. Moreover, several studies (9, 19–21) have demonstrated that the overall prevalence of headaches has shown a supplementary rise within the same age group when compared to previous data.

However, Özge et al. (22) conducted a study focusing on children and found no significant association between age and the increase in headache prevalence. This finding is interesting and suggests that age may not be the sole determining factor in the prevalence of headaches among children. Furthermore, Shivpuri et al. (23) reported a decrease in the prevalence of headaches among school-aged children aged 11 to 15. This finding contradicts the general trend observed in the previous studies and highlights the need for further investigation into the factors influencing headache prevalence in this specific age group.

Regarding the prevalence of tension headaches in urban or rural areas, our own results indicate that there is no significant difference in the occurrence of headaches among students in urban and rural areas. However, a separate study conducted by Togha et al. in Iran reveals that the prevalence of headaches is higher in urban areas compared to rural areas (14). Furthermore, a meta-analysis incorporating data from 14 studies suggests that, in general, tension headaches are less prevalent in rural areas when compared to urban areas (24). Another study in Egypt showed higher frequency of TTH in rural residents (25). As the results are not consistent in different studies, it might be due to geographic, cultural, nutritional, economic and other factors. It is plausible to consider that this discrepancy may be attributed to differences in the sampling methods employed.

Our study further reveals that a significant majority, exceeding 70% of participants, experience headache episodes lasting less than 2 h across all three groups: low frequency, high frequency, and chronic. In their study, Wo¨ber-Bingol et al. (26) have presented the assertion that an increase in the diagnosis of migraines is accompanied by a reduction in the duration of headaches in children. This finding has been corroborated by several other studies (27–30). Conversely, Abu-Arafeh et al. (31) have reported that the duration of headache attacks may exhibit variability within an individual. In the same way, Poyrazog˘lu et al. (9) have reached the conclusion that the typical duration of tension-type headache attacks ranges from 0.5 to 1 h in most cases.

In this study, participants consistently described tension headaches as primarily manifesting as a pressing sensation, affecting both sides of the head. Interestingly, symptoms such as nausea, vomiting, and photophobia were not frequently reported by the participants; however, 78.1% of our patients experienced phonophobia, that is higher than what reported by others. Poyrazog˘lu et al. (9) conducted a study in which they found that children with TTHs did not commonly report experiencing nausea and vomiting. Nevertheless, it is important to acknowledge that photophobia and phonophobia can be prevalent symptoms, as they were reported in 15.9% of these children. Ashina et al. reported phonophobia in 18.1% of their patients diagnosed with episodic TTH (32). In another study by Köseoglu E et al., phonophobia was the most common concomitant symptom (50.9%) of TTH (33).

The study findings shed light on the profound impact that tension headaches have on various aspects of individuals’ daily lives. As the number of headache days escalates, participants have reported an increased number of absences from both school and work. Consequently, this has led to a rise in the number of parental leave days taken by caregivers in order to address their children’s condition. Along the same line in a study conducted by Wöber-Bingöl et al., the researchers have successfully demonstrated the correlation between the prevalence of headaches in children and the subsequent rise in school absenteeism rates or the inability to actively participate in school activities. Additionally, it has been observed that parents of children suffering from headaches are compelled to take at least 1 day off from work per month (34).

In this study, we observed that the average score for quality of life was found to be highest among individuals who did not experience headaches. Similarly, the research conducted by Wöber-Bingöl et al. has provided evidence that headaches in children can significantly impact their quality of life. Through statistical analysis, an inverse association between the severity of the headache and the overall quality of life was observed in this study (34). In addition to academic problems, headaches in children can also have a negative effect on family relationships, participation in social activities, making friends, and the psychological state.

## Conclusion

The high prevalence of TTHs and their detrimental effects on children’s and adolescents’ daily activities emphasize the importance of effective management strategies and interventions. It is crucial to develop approaches that can alleviate the burden of this condition on individuals lives of these age groups. Further research in this area may help to better understand the underlying factors contributing to the occurrence of TTH in this population.

## Data availability statement

The original contributions presented in the study are included in the article/supplementary material, further inquiries can be directed to the corresponding author.

## Ethics statement

The studies involving humans were approved by The study received the esteemed approval of the Ethics Committee of the Iranian Center of Neurological Research, under the ethical code IR.TUMS.NI.REC.1397.211. The studies were conducted in accordance with the local legislation and institutional requirements. Written informed consent for participation in this study was provided by the participants’ legal guardians/next of kin. Written informed consent was obtained from the individual(s), and minor(s)’ legal guardian/next of kin, for the publication of any potentially identifiable images or data included in this article.

## Author contributions

MT: Conceptualization, Data curation, Project administration, Supervision, Writing – original draft, Writing – review and editing, Funding acquisition, Methodology. EJ: Data curation, Writing – original draft. ZS: Data curation, Writing – review and editing. KK: Formal analysis, Writing – review and editing. HM: Writing – review and editing. MM: Writing – review and editing. SB: Data curation, Writing – review and editing.

## Funding

The author(s) declare that no financial support was received for the research, authorship, and/or publication of this article.

## Conflict of interest

The authors declare that the research was conducted in the absence of any commercial or financial relationships that could be construed as a potential conflict of interest.

## Publisher’s note

All claims expressed in this article are solely those of the authors and do not necessarily represent those of their affiliated organizations, or those of the publisher, the editors and the reviewers. Any product that may be evaluated in this article, or claim that may be made by its manufacturer, is not guaranteed or endorsed by the publisher.
